# Effect of magnesium supplementation on pregnancy outcome in gestational diabetes mellitus patients: A meta‐analysis of randomized controlled trials

**DOI:** 10.1002/fsn3.2561

**Published:** 2022-09-07

**Authors:** Qiuchan Qu, Rong Rong, Jinhua Yu

**Affiliations:** ^1^ Department of Reproductive Health, Maternal and Child Health Hospital Affiliated to Nantong University Nantong China

**Keywords:** gestational diabetes mellitus, magnesium supplementation, meta‐analysis, pregnancy, randomized controlled trial

## Abstract

Conflicting evidence exists regarding the effectiveness of magnesium supplementation during pregnancy in gestational diabetes mellitus (GDM) patients. This meta‐analysis examines the effect of magnesium on glycemic indices and metabolic status in GDM. We searched databases for randomized controlled trials (RCTs) conducted, and after applying inclusion and exclusion criteria, a total of four RCTs were considered eligible for the analysis. Outcome parameters included markers for glycemic control and metabolic status. A total of four RCTs with 198 participants (control = 99; magnesium supplemented = 99) were selected for the analysis. Magnesium supplementation resulted in a significant reduction in markers of glycemic control—fasting plasma glucose (standard mean difference ($\hat{\mu }$) = −0.83; 95% CI: [−1.13, –0.54]; *p*‐value <.0001), and insulin levels ($\hat{\mu }$ = −0.95; 95% CI: [−1.38, −0.52]; *p*‐value <.0001). Also, Mg intake resulted in altered oxidative stress markers TAC ($\hat{\mu }$ = 1.09; 95% CI: [0.10, 2.07]; *p*‐value = .03) of the pregnant women. No significant effect on GSH and CRP levels was observed. This study provides evidence of the positive effects of magnesium intervention on insulin sensitivity and oxidative stress in GDM patients.

## Graphical abstract

Meta‐analysis on the effect of Mg on glycaemic indices in GDM patients is reported. Outcome parameters include markers for glycaemic control and metabolic status. Four RCTs were after applying inclusion and exclusion criteria for the analysis. Mg intake altered oxidative stress marker, inflammation, cholesterol and VLDL.

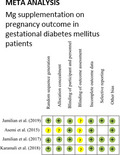

## 1 INTRODUCTION

Gestational diabetes mellitus (GDM) is defined as any degree of glucose impairment with onset or first recognition during pregnancy (Agarwal et al., 2015). It affects approximately 18% of women worldwide and is associated with a high mortality and morbidity rate (Singh et al., 2015). The significant increase in the prevalence of GDM in the past decade is primarily attributed to a sedentary lifestyle, obesity, and increased maternal age (Ferrara, 2007; Karaçam & Çelik, 2021; Larrabure‐Torrealva et al., 2018). Also, the wide discrepancy in diagnostic criteria of GDM often leads to statistical misrepresentation (Alberico et al., 2004). GDM results in pregnancy complications and is known to adversely affect maternal and neonatal outcomes. While the females with GDM are at an increased risk of developing pre‐eclampsia, labor abnormalities, cardiovascular, and other metabolic disorders, the neonates may suffer from macrosomia, hyperbilirubinemia, low birth weight, and hypoglycemia (Koivunen et al., 2020; Murray & Reynolds, 2020; Savage et al., 2020; Wendland et al., 2012). The massive impact of both short‐term and long‐term effects of GDM on maternal and neonatal health necessitates the proper management of the disease during pregnancy. The insulin resistance and pancreatic β cell impairment result in increased levels of blood sugar and subsequent development of GDM (Kaaja & Rönnemaa, 2008). To maintain blood glucose homeostasis, insulin therapy and anti‐diabetic agents are commonly used; however, their use during pregnancy is reported to exhibit severe side effects and thus remain controversial (Coustan & Lewis, 1978; Kalra et al., 2015). In addition, insulin resistance is associated with high levels of inflammatory and oxidative stress markers in GDM patients.

Nutritional interventions in form of dietary supplements and modifications are gaining popularity for the prevention and management of GDM. Various studies have established the role of low glycemic, high fat, and a balanced nutrient diet in decreasing the risk of GDM development in pregnant women (Jin et al., 2020; Shin et al., 2015; Spencer et al., 2015; Zhang et al., 2006). The positive effects of vitamins, minerals, fatty acids, and probiotics have been intensively studied in several clinical trials (D’Anna et al., 2013; Looman et al., 2019; Plows et al., 2019; Saldana et al., 2004; Shin et al., 2015). The promising ability of micro‐ and macronutrients to modulate glucose tolerance and improve neonatal and maternal parameters such as oxidative stress, inflammation, and insulin sensitivity makes them an ideal and safe intervention to be used in pregnant females.

Magnesium (Mg), an important micronutrient, plays a critical role in nucleic acids and protein synthesis, cell metabolism, cell replication, glucose regulation, and insulin signaling. In turn, insulin acts as an important regulatory factor intracellular magnesium accumulation (Paolisso et al., 1990). Mg is widely implicated as a causative factor in diabetes, hypertension, cardiovascular disorders, and neuromuscular manifestations (Swaminathan, 2003). Low serum Mg is often correlated with high insulin resistance in diabetic patients (Huerta et al., 2005; Kahil et al., 1966).

Gestational diabetes mellitus is frequently accompanied by hypomagnesemia, a state of Mg deficiency associated with pregnancy‐related complications. A reduction in total cellular Mg levels is reported in GDM while serum Mg concentration remains debatable (Musavi et al., 2019; Nabouli et al., 2016; Wang et al., 2002). Moreover, the significant alteration in levels of albumin and creatinine factors is responsible for maintaining Mg homeostasis. Serum Mg is also suggested as a reliable marker to predict the post‐partum transition of GDM to type 2 diabetes mellitus (Naser et al., 2019). Limited clinical trials investigating the role of serum Mg in GDM development have reported contradictory findings, probably due to the difference in gestational age and the dietary intake of Mg in the subjects. Despite the paucity of Mg studies in GDM, the supplementation of Mg during pregnancy is suggested to improve maternal and neonatal outcomes due to the beneficial effects observed in type 1 diabetes patients after oral Mg intake (Rodríguez‐Morán & Guerrero‐Romero, 2003; Veronese et al., 2016). In addition to the insulin signaling, Mg is also known to influence systemic inflammation, antioxidant parameters, and lipid profile in diabetes patients (Jansen van Vuuren et al., 2019; Kim et al., 2010; Nasri & Baradaran, 2008; Olatunji & Soladoye, 2007). However, the effect of Mg on these processes in GDM remains unclear and there seems to be no consensus among researchers regarding the use of Mg supplements in pregnant women. A meta‐analysis helps in finding an association between different factors to comprehensively understand the results obtained until now using statistical tools. Thus, in the present study, we have used a meta‐analysis of RCTs to assess the effect of Mg supplementation in GDM patients during pregnancy on (1) glycemic parameters—Fasting plasma glucose (FPG); Insulin levels; Homeostatic Model Assessment of Insulin Resistance (HOMA‐IR), (2) biomarkers of metabolic status including oxidative stress, and inflammation—Glutathione (GSH), Total antioxidant capacity (TAC), (3) Inflammation biomarker—C‐reactive protein (CRP). These markers are chosen on the basis of previous literature reports that have established a relationship between GDM pathogenesis and the level of these markers (Alptekin et al., 2016; Jansen van Vuuren et al., 2019; Kim et al., 2010; Nasri & Baradaran, 2008; Olatunji & Soladoye, 2007; Zhu et al., 2015).

## 2 MATERIALS AND METHODS

This meta‐analysis was conducted following the Preferred Reporting Items for Systematic Reviews and Meta‐Analyses (PRISMA) guidelines (Liberati et al., 2009).

### 2.1 Search strategy

A comprehensive literature search in electronic databases PubMed and Cochrane Library was performed to identify eligible trials. The main search words for article texts, abstract, or MeSH headings were as follows: magnesium, magnesium supplementation, gestational diabetes mellitus, pregnant women, randomized controlled trial, clinical trial, and intervention. These keywords were combined using the Boolean logic operator AND to refine search criteria. The databases were searched for articles from the time of inception to 31st June 2021. Additional articles were searched on the clinical trials registry or using bibliography of the retrieved articles. The language of the searched articles was restricted to English.

### 2.2 Eligibility criteria

Two authors, Q.Q. and R.R. independently screened the records.

Inclusion criteria were as follows: (1) pregnant women with confirmed GDM; (2) randomized clinical trials; (3) the presence of placebo or control group; (4) subjects aged 18–40 years; (5) oral magnesium alone or in combination with other supplements; (6) studies in English.

Exclusion criteria were as follows: (1) animal studies; (2) pregnant women without confirmed GDM; (3) studies conducted post‐partum; (4) studies not reporting FPG values after Mg supplementation; (5) women with pre‐existing diabetes condition (type 1 or 2); (6) Clinical trials studying the preventive or protective effect of magnesium supplementation on GDM development.

### 2.3 Data extraction

Independent extraction of articles by two authors, Q.Q. and R.R., was carried out using inclusion and exclusion criteria followed by data inspection for each paper. The full text of eligible studies was retrieved. All the included studies were checked for (1) design of the study to reduce the biases in effect sizes; (2) measure of the change in outcome factors as an effect of magnesium; (3) demographic characteristics or correlating factors for the effect of magnesium.

The following information from each study was extracted: authors and year of publication, country of origin, sample size, study type, and participants’ characteristics—age, body mass index (BMI) at baseline, type of intervention, dosage, duration, and outcome assessed post‐intervention.

Primary outcomes were restricted to glycemic control including 1. FPG; 2. Insulin levels; 3. HOMA‐IR. Secondary outcomes included 1. Oxidative stress biomarkers—GSH, TAC, 2. Inflammation biomarker—CRP. Data were represented in the form of mean ± standard deviation (*SD*) for each of the outcome variables, and as mean for mean age and BMI at baseline for the cohorts in each of the studies.

### 2.4 Quality assessment

Two authors, Q.Q. and R.R., independently assessed the quality of selected RCTs based on Cochrane collaboration in the following aspects: selection bias, performance bias, attrition bias, reporting bias, decision bias, and any other bias (Liberati et al., 2009). These were categorized into low risk (if adequate information is available), unclear risk (not adequate information), and high risk (potential influence on the outcomes). Disagreements were resolved by consensus. The assessment was based on the information provided in the published papers only.

### 2.5 Data analysis

The influence of magnesium supplementation on different outcome variables was evaluated using the meta‐analysis. Since characteristics of participants and type of intervention were different in all studies, a random effect model was utilized to perform statistical analysis. The strength and association between the outcome variables and Mg supplementation were assessed using a standardized mean difference with 95% confidence interval (CI). In case of a change in mean and standard deviation (*SD*) for outcome variables not being reported, the change was computed in mean as a difference of (reported mean at in follow‐up in the treated group—reported mean at baseline in treated group) and (reported mean at follow‐up in the control group – reported mean at baseline in the control group). For standard deviation, we used the following formula—*SD* = standard error (*SE*) *square root (*n*), where n denotes the total number of subjects. For studies not reporting *SE*, we referred to section “6.5.2.3 – Obtaining standard deviations from standard errors, confidence intervals, *t* statistics and *P* values for differences in means” of Cochrane Handbook for Systematic Reviews of Intervention (Higgins et al., 2019). Additionally, the conversion factor (CF) was applied in all the cases where values were not provided in standard units in any study.

1. FPG: mmol/L to mg/dl by using CF 18.018.
2. Insulin: 1 μIU/ml = 6.00 pmol/L (Knopp et al., 2019).

Positive effect size reveals an increase in biomarker levels with magnesium supplementation and vice versa. Study heterogeneity among the studies was evaluated using the Cochran *Q* test and *I* ^2^ index. *I* ^2^ values of 25%, 50%, and 75% corresponded to different levels of heterogeneity with 25% being the lowest (Higgins & Thompson, 2002). R statistical software was used to perform all the analyses and plot the graphs in the paper. Forest plots were plotted for meta‐analysis. The horizontal line corresponding to each study [author (year)] represents the contribution to the overall model. The black square on the horizontal line shows the weight of the study or contribution it had to the overall analysis. Larger the box, the larger the weight of the study in the meta‐analysis. The horizontal line to both sides of the black box represents the 95% CI. The red polygon shows the estimate of the model along with 95% CI.

### 2.6 Meta‐regression

Meta‐regression was performed using a mixed‐effect model. It was used to identify the association between outcome parameters and study patient characteristics. We assessed the role of moderator on the overall estimate of the effect size of the outcome biomarker. Publication bias using the funnel plot and Begg’s adjusted rank correlation test could not be performed as the total number of studies for the required outcome of interest did not exceed ten. Forest plot, graphical representation of meta‐analysis, has been used to understand the overall effect size of the studies included.

## 3 RESULTS

### 3.1 Study selection

**FIGURE 1:**
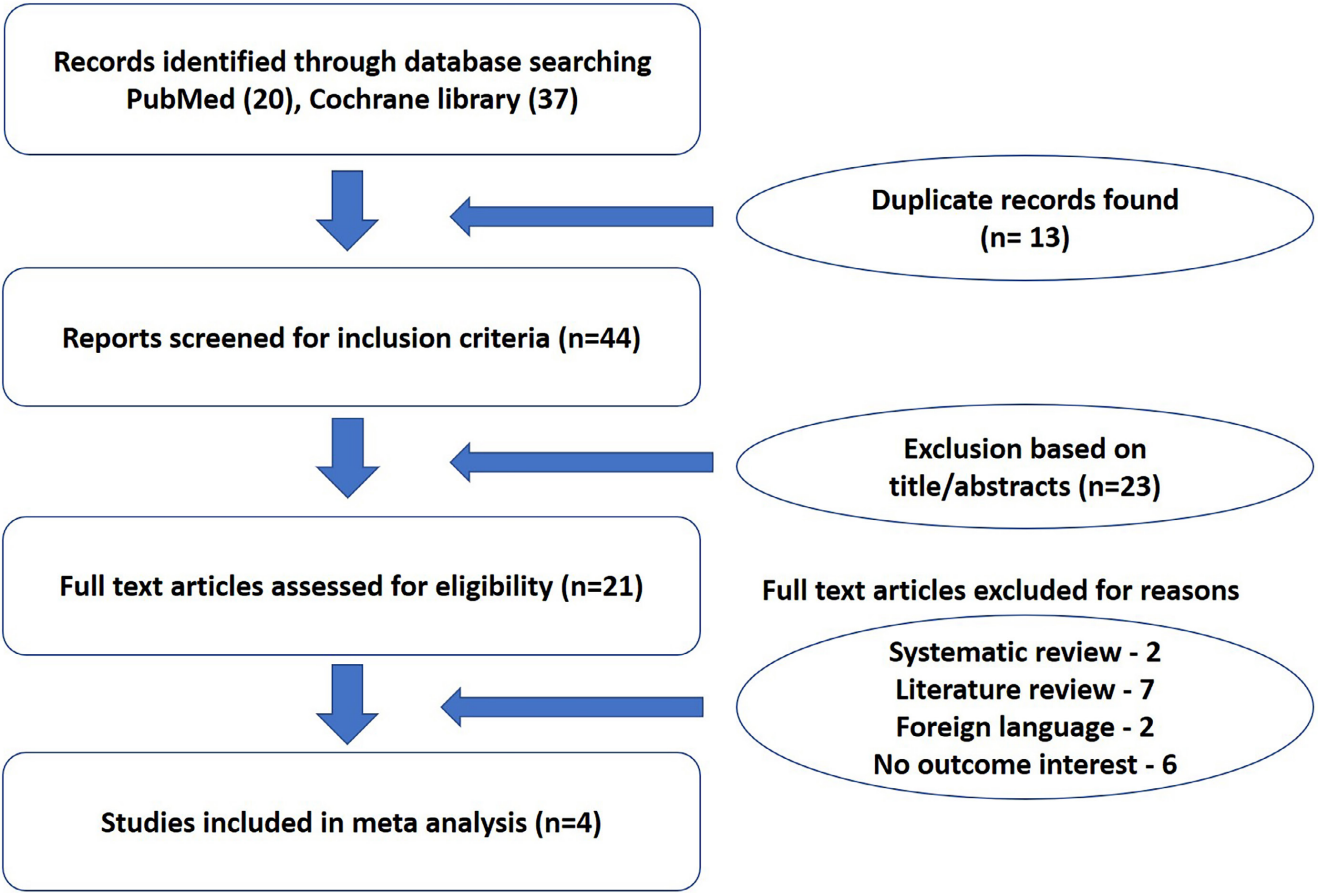
Flowchart for preferred reporting items for systematic reviews and meta‐analyses (PRISMA) study selection

Initially, after the PubMed and Cochrane Library database search, 57 published studies were identified and the abstracts were reviewed. For the PubMed database, filters such as randomized control trial, clinical trial, clinical trial phase I, II, and clinical study, clinical protocol were applied. Initially, 13 records were found to be duplicate and removed. Furthermore, on screening after reading tittle and abstract, 23 records were excluded based on exclusion criteria. Of the 21 extracted records, reviews were excluded. Finally, four studies were found to be eligible from both databases and included for the meta‐analysis. The detailed process of the search strategy is presented in Figure 1. A total of 198 sample size subjects were recruited from four RCTs. Included studies were published between 1994 and 2020.

### 3.2 Study characteristics

A total of four RCTs with 198 participants were selected for this analysis (Asemi et al., 2013; Jamilian et al., 2017, 2019; Karamali et al., 2018). The baseline characteristics of the studies are summarized in Tables 1 and 2. All the four trials were conducted in Iran during 2011–2019 with an intervention group consisting of pregnant females diagnosed with GDM. American Diabetes Association guidelines for GDM diagnosis were followed by all the studies. The intervention period varied from 4 to 6 weeks, and the dosage of Mg varied from 200–250 mg. Out of four trials included, three used Mg in combination with other supplements, including the DASH diet, vitamin D, calcium, and vitamin E. Outcome measures varied across studies with glycemic parameters, that is, FPG, insulin, and HOMA‐IR being most reported. In addition, oxidative stress and inflammation biomarkers were also reported in few studies (Asemi et al., 2013; Jamilian et al., 2019).

**TABLE 1:** Clinical characteristics of the subjects at the baseline

Study	Country	Study type	Sample size Total (CG, IG)	Maternal age (years)		BMI	
				IG	CG	IG	CG
Asemi et al. (2013)	Iran	Randomized, placebo controlled parallel	38 (19, 19)	27.7 ± 5.4	29.7 ± 5.6	30.2 ± 4.6	29.7 ± 3.3
Jamilian et al. (2017)	Iran	Randomized, double blind, placebo controlled	40 (20, 20)	27.8 ± 3.4	27.1 ± 4.9	26.1 ± 1.9	27.4 ± 3.2
Karamali et al. (2018)	Iran	Randomized, double blind, placebo controlled	60 (30, 0)	30.0 ± 4.5	31.1 ± 4.2	27.4 ± 4.8	27.0 ± 2.6
Jamilian et al. (2019)	Iran	Randomized, double blind, placebo controlled	60 (30, 30)	27.7 ± 4.0	29.1 ± 4.1	25.8 ± 3.7	25.3 ± 2.5

Abbreviations: CG, control group; IG, intervention group.

**TABLE 2:** Details of the studies included

Study	Duration (weeks)	Intervention			Study date	Outcome variables assessed that are used in this analysis
		Type	Dose (mg/day)	Control group		
Asemi et al. (2013)	4	Dietary (DASH diet)	Not specified^a^	Yes	April 2011–Dec 2011	FPG, insulin, HOMA‐IR, CRP, TAC, and GSH
Jamilian et al. (2017)	6	Magnesium oxide	250	Yes	Mar 2017–Jun 2017	FPG
Karamali et al. (2018)	6	Magnesium with other supplements	200	Yes	April 2017–Jun 2017	FPG, insulin, HOMA‐IR, and lipid profile
Jamilian et al. (2019)	6	Magnesium with other supplements	200	Yes	Mar 2017–Nov 2017	FPG, TAC, GSH, and CRP

^a^ Mg intake in diet given as mean ± *SD*: control diet‐272 ± 45.9; DASH diet‐363.9 ± 14.2.

The details of the additional supplements used in the studies are given below:

In the study conducted by Asemi et al. (2013), Dietary Approaches to Stop Hypertension (DASH) diet plan was followed in GDM patients. DASH diet is rich in fruits, vegetables, whole grains, low‐fat dairy products, and was low in saturated fats, cholesterol, refined grains, and sweets. Restricted amount of sodium intake was <2000 mg/day (Asemi et al., 2013).

Other studies reported that women with GDM received 100 mg magnesium, 4 mg zinc, and 400 mg calcium plus 200 IU vitamin D supplements as compared to placebo twice a day for 6 weeks (Jamilian et al., 2019; Karamali et al., 2018).

### 3.3 Evaluation of risk bias

**FIGURE 2:**
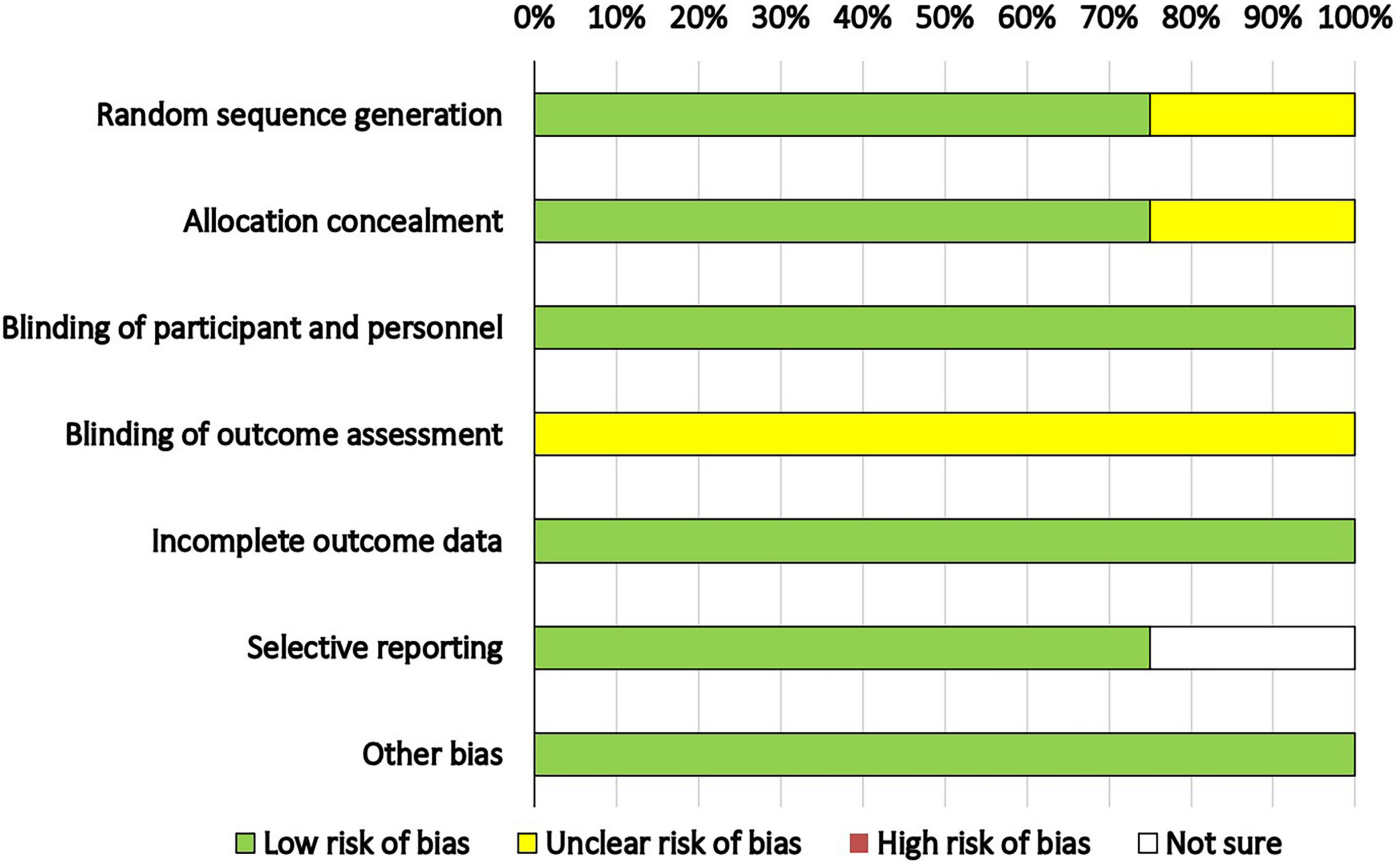
Risk‐of‐bias graph per type of bias assessed. Random sequence generation—75% (low risk of bias). Allocation concealment—75%. Incomplete outcome data—100% (low risk of bias). Selective reporting—low risk of bias‐75%, white 25%

**FIGURE 3:**
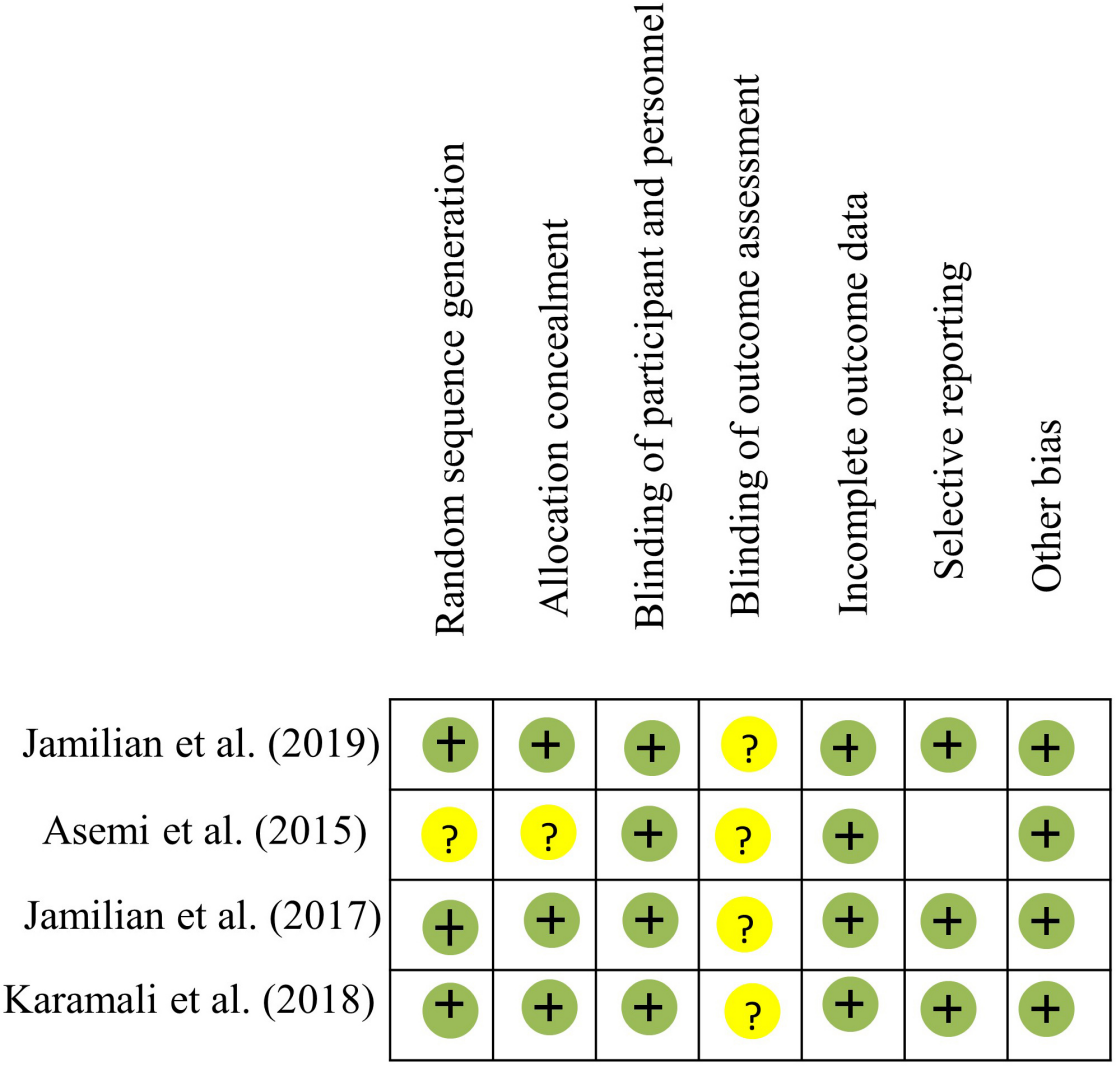
Risk‐of‐bias summary for the studies; assessment of risk of bias for each trial using the Cochrane risk‐of‐bias tool. Green symbols correspond to low risk of bias, yellow to unclear risk of bias, and red to high risk of bias

A summary of risk bias evaluated in the included studies is illustrated in Figures 2 and 3. A low risk of selection bias was found in all studies (computer‐generated allocation). Low risk for performance bias and reporting bias was found in all the studies. A high risk for attrition bias in a study by Asemi et al. (2013) was found due to a high rate of loss which is likely to influence the outcome (Asemi et al., 2013). No reporting bias was observed. Publication bias could not be evaluated due to a small number of studies.

- Effect of Mg supplementation on
- Glycemic control markers

The overall impact of magnesium supplementation on FPG, insulin, and HOMA‐IR was assessed in 4, 2, and 2 studies, respectively. For FPG, insulin and HOMA‐IR the random effects had less heterogeneity with *I* ^2^ = 0% which indicates that all the studies observed similar effects of Mg supplementation on glycemic markers and thus, the Mg intake could significantly reduce the insulin levels.

**FIGURE 4:**
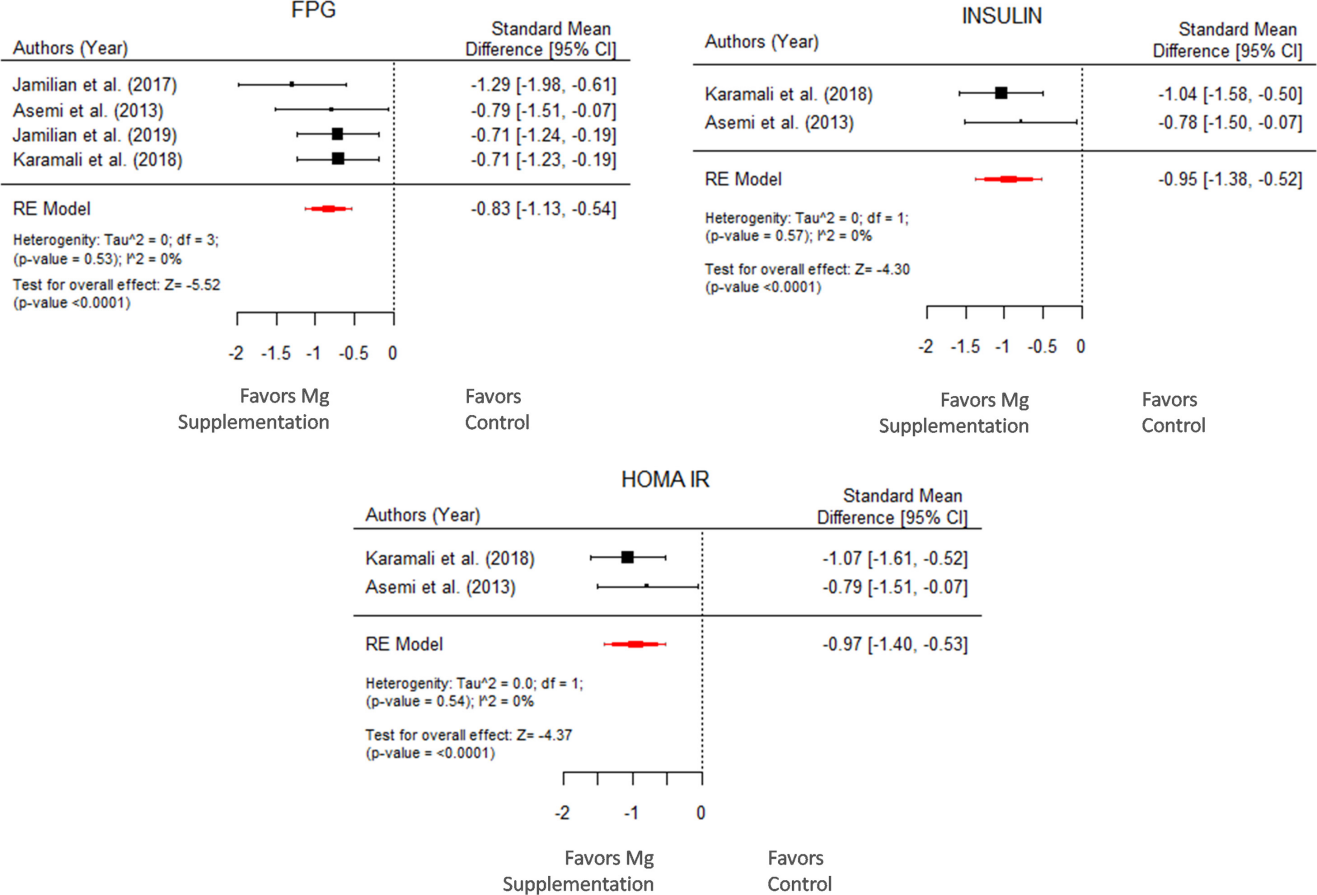
Forest plot displaying standard mean difference and 95% CI for the impact of Mg supplementation on glycemic parameters

Figure 4 shows a forest plot with the effect of magnesium supplementation on various glycemic parameters. Cohort treated with magnesium supplements had a significant effect on FPG levels ($\hat{\mu }$ = −0.83 with 95% CI: −1.13 to –0.54 and *p*‐value <.0001) as well as on insulin levels ($\hat{\mu }$ = −0.95 with 95% CI: −1.38 to −0.52 and *p*‐value <.0001). The model results suggest that the intake of magnesium supplements by the pregnant woman is beneficial to reduce the FPG level and insulin level. Similar effect was observed for magnesium supplementation on HOMA‐IR levels ($\hat{\mu }$ = −0.97 with 95% CI: −1.40 to −0.53 and *p*‐value <.0001).

- Oxidative stress parameters

**FIGURE 5:**
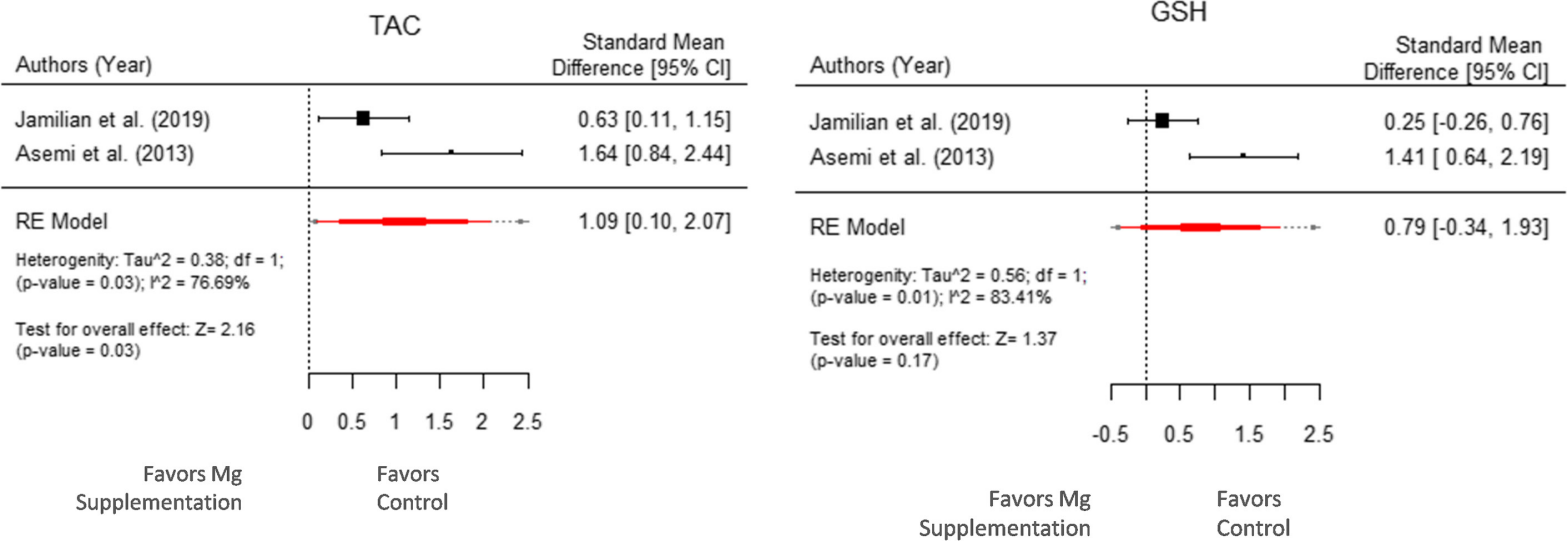
Forest plot displaying standard mean difference and 95% CI for the impact of Mg supplementation on oxidative stress parameters

We had only two studies for oxidative stress parameters—TAC and GSH. MDA had only 1 study and was excluded for meta‐analysis. The heterogeneity was high for TAC and GSH with *I* ^2^ > 75%. A high *I* ^2^ value indicates the variability observed in the results across different studies. While the random effect model (Figure 5) showed a significant effect of Mg supplementation on TAC ($\hat{\mu }$ = 1.09 with 95% CI: 0.10 to 2.07 and *p*‐value = .03), no such effect of Mg supplementation on GSH ($\hat{\mu }$ = 0.79 with 95% CI: −0.34 to 1.93 and *p*‐value = .17) was observed.

- Inflammation markers

**FIGURE 6:**
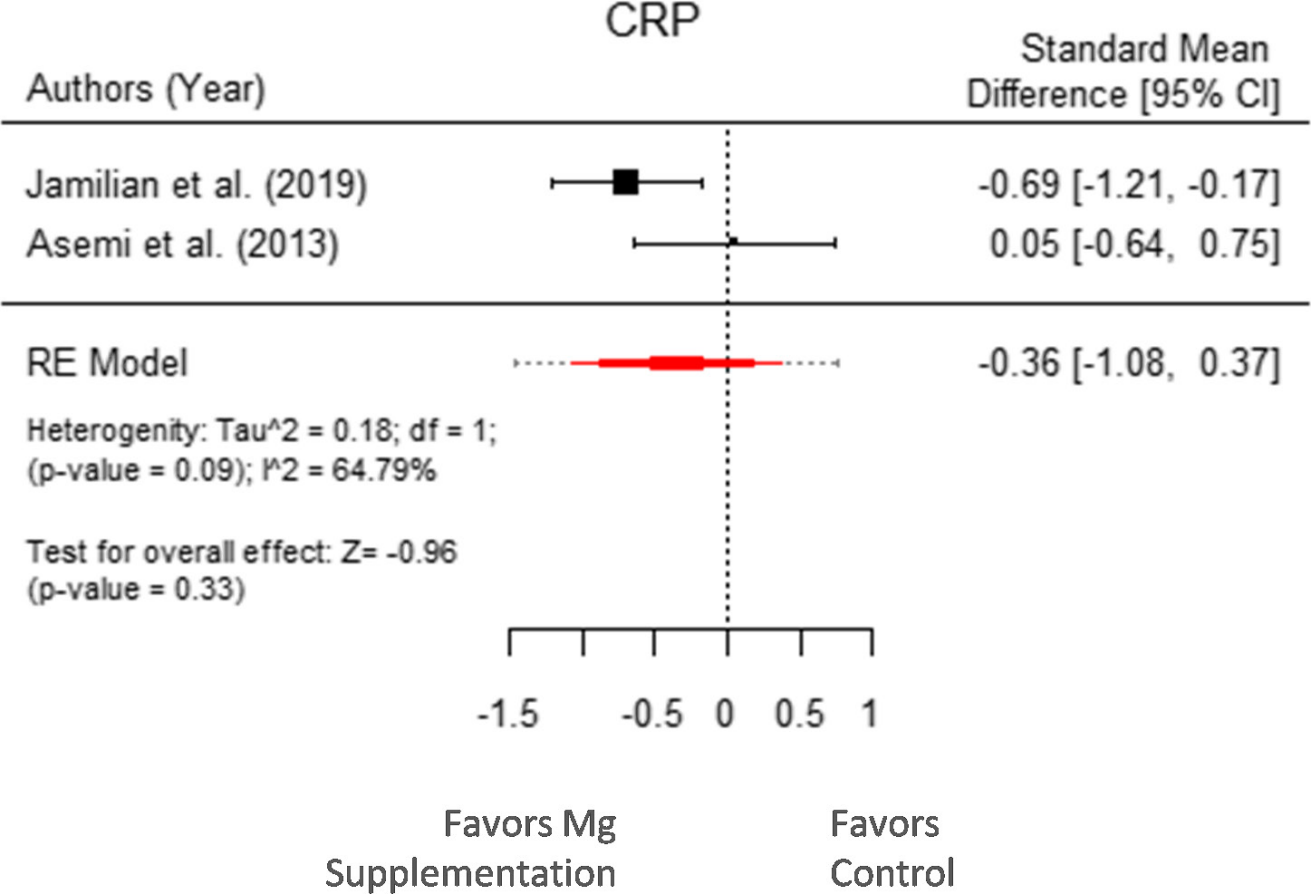
Forest plot displaying standard mean difference and 95% CI for the impact of Mg supplementation on inflammation parameter

Figure 6 shows the result for forest plot with random effect model resulting in no significant effect on Mg supplementation on CRP marker of inflammation ($\hat{\mu }$ = −0.36 with 95% CI: −1.08 to 0.37 and *p*‐value = .33). The heterogeneity factor was 0.18 with *I* ^2^ = 64.79% (*p*‐value = .09). Thus, the Mg supplements did not exert any effect on GDM‐associated inflammation.

### 3.4 Meta‐regression

Meta‐regression was carried out to check the effect of moderators—BMI at baseline and Mg supplementation. Meta‐regression analysis (Table 3) resulted in no influence of BMI at baseline (levels: <27and ≥27) for any significant difference in outcome parameters.

**TABLE 3:** Meta‐regression analysis for BMI at baseline as moderator

	BMI at baseline			
	Coefficient	*SE*	*p*‐Value	95% CI
FPG	0.19	0.30	.52	−0.40 to 0.78
TAC	0.24	1.23	.84	−2.18 to 2.67
GSH	0.21	1.57	.88	−2.86 to 3.29
CRP	0.41	0.43	.33	−0.43 to 1.26

We also wanted to check whether there was any effect on outcome parameters when supplementation of Mg was either given to the treated cohort alone or along with other supplementation. There was a highly significant difference when Mg was given in addition to other supplementation to the treated group on HOMA‐IR (*p* < .001) as compared to other outcome parameters (Table 4).

**TABLE 4:** Meta‐regression analysis for Mg + additional supplementation as moderator

	Mg + additional supplementation			
	Coefficient	*SE*	*p*‐Value	95% CI
FPG	0.56	0.38	.14	−0.19 to −0.61
INSULIN	0.17	0.45	.69	−0.71 to 1.07
HOMA‐IR	−1.48	0.43	.0005	−2.33 to −0.64
TAC	0.89	0.83	.28	−0.73 to 2.53
GSH	1.21	0.977	.21	−0.70 to 3.12
CRP	0.12	0.61	.83	−1.07 to 1.32

## 4 DISCUSSION

Mg deficiency during pregnancy is associated with the development of GDM, hypertensive disorders, preterm labor, and adverse neonatal outcomes (Almonte et al., 1999; He et al., 2016). A meta‐analysis of dietary magnesium supplementation in healthy pregnant females indicated no significant effect on maternal and fetal outcomes (Makrides et al., 2014). Nonetheless, the low quality of evidence and high bias in the studies was likely to influence the outcomes assessed. The dietary intervention during pregnancy is also considered both a preventive and protective strategy against adverse maternal outcomes (Coustan, 2013; Fang et al., 2016; Saldana et al., 2004; Shin et al., 2015). Thus, the present study was conducted to examine the relationship between Mg intake and reduction in GDM severity by analyzing markers of glycemic control, oxidative stress, and inflammation. From the data extracted from four RCTs included in this study, it was found that there was a significant improvement in glucose metabolism and insulin sensitivity (FPG, insulin) in addition to specific marker of oxidative stress TAC, with no considerable effect on inflammation (CRP) and other markers like HOMA‐IR, and GSH (Asemi et al., 2013; Jamilian et al., 2017, 2019; Karamali et al., 2018). In addition, the meta‐regression study further revealed that the use of co‐supplements with Mg significantly affected HOMA‐IR, with no effect on any other outcome parameters (Table 4). This could be due to the dietary modifications that can modulate HOMA‐IR values. BMI factor did not play any role in modulating the effect of the Mg intervention.

The dysregulated insulin signaling in GDM results in hyperglycemia that subsequently leads to increased inflammation, oxidative stress, and hyperlipidemia. The high levels of pro‐inflammatory cytokines and oxidative indices such as MDA are associated with an imbalance between the pro‐oxidant and antioxidant systems in GDM patients (Genc et al., 2017). In fact, inflammation marker CRP along with Mg has been shown to accurately predict GDM development (Naser et al., 2019). Clinical trials have also established the effects of Mg on oxidative stress and inflammation in other gestational disorders like pre‐eclampsia (Abad et al., 2015). Moreover, the earlier systematic reviews have suggested the important role of magnesium supplementation in reducing the magnitude of diabetes‐related complications by regulating glucose levels and insulin sensitivity (Fang et al., 2016; Verma & Garg, 2017; Veronese et al., 2016).

Overall, the results of the present study are in accordance with the literature and provide an overview of the possible therapeutic role of Mg in GDM by targeting different processes related to it. It is to be that although we did not find a significant association of Mg intake on all the biomarkers tested, the effect on critical parameters like FPG, insulin, and TAC paves the way for future mechanistic studies.

## 5 STRENGTHS AND LIMITATIONS OF THE STUDY

The population‐based evidence regarding the effect of dietary Mg on GDM is lacking. To our knowledge, this is the first study reporting the effect of Mg supplementation in pregnant women with GDM. The inclusion of only RCTs strengthened the study since all studies except one followed high standards of randomization and were associated with reduced risk of bias. The similar baseline characteristics, that is, age, BMI, and country of origin, reduced the potential confounding from demographic and other factors.

The study is potentially limited by the low number of studies included and the small sample size which may have affected the result interpretation. The use of co‐supplements with Mg could have acted as confounding factors, and their in‐depth potential on all the outcome parameters could not be evaluated due to the high variability in the study design, the low number of studies, and lack of missing information, though a significant effect on FPG and HOMA‐IR was obtained. All studies included were conducted in a single country, and thus, the conclusion may not apply to other races or ethnicity. The restriction to the English language limited the number of studies. Lack of publication bias and potential influencing factors could not be assessed. Thus, more robust clinical trials involving a higher number of participants with gestation matched controls are required to establish concrete evidence regarding the role of Mg supplementation in GDM patients.

## 6 CONCLUSIONS

Results from this meta‐analysis prove the beneficial effects of magnesium supplementation in pregnant women with GDM. It is suggested that clinical trials with large sample size and specifically with oral magnesium supplements should be conducted to gain a better understanding of the effect on pregnancy outcomes.

## CONFLICT OF INTEREST

The authors declare that there is no conflict of interest.

## AUTHOR CONTRIBUTIONS

**Qiuchan Qu:** Conceptualization (equal); Formal analysis (equal); Investigation (equal); Methodology (equal); Resources (equal); Validation (equal); Visualization (equal); Writing‐original draft (equal). **Rong Rong:** Conceptualization (equal); Formal analysis (equal); Investigation (equal); Methodology (equal); Resources (equal); Validation (equal); Visualization (equal); Writing‐original draft (equal). **Jinhua Yu:** Conceptualization (equal); Methodology (equal); Resources (equal); Supervision (equal); Writing‐review & editing (equal).

## ETHICAL APPROVAL

Studies involving human subjects: No ethical conflicts are involved in this project as this work is a meta‐analysis research of already published data. Studies involving animal or human subjects: No ethical conflicts are involved in this project as this work is a meta‐analysis research of already published data.

## Data Availability

The data used in this meta‐analysis shall be made available upon request.
